# Object recognition and localization from 3D point clouds by maximum-likelihood estimation

**DOI:** 10.1098/rsos.160693

**Published:** 2017-08-16

**Authors:** Harshana G. Dantanarayana, Jonathan M. Huntley

**Affiliations:** Loughborough University, Wolfson School of Mechanical, Electrical and Manufacturing Engineering, Loughborough, Leicestershire, LE11 3TU, UK

**Keywords:** fringe projection 3D scanning, pose estimation, object recognition, industrial inspection

## Abstract

We present an algorithm based on maximum-likelihood analysis for the automated recognition of objects, and estimation of their pose, from 3D point clouds. Surfaces segmented from depth images are used as the features, unlike ‘interest point’-based algorithms which normally discard such data. Compared to the 6D Hough transform, it has negligible memory requirements, and is computationally efficient compared to iterative closest point algorithms. The same method is applicable to both the initial recognition/pose estimation problem as well as subsequent pose refinement through appropriate choice of the dispersion of the probability density functions. This single unified approach therefore avoids the usual requirement for different algorithms for these two tasks. In addition to the theoretical description, a simple 2 degrees of freedom (d.f.) example is given, followed by a full 6 d.f. analysis of 3D point cloud data from a cluttered scene acquired by a projected fringe-based scanner, which demonstrated an RMS alignment error as low as 0.3 mm.

## 1. Introduction

The recognition and localization of objects is one of the most complex problems in machine vision. Different constraints imposed by its applications have led to various interpretations of the problem. We consider in this paper the recognition and localization of a known object or objects, with possible multiple instances, using single or multiple quasi-three-dimensional (2.5D or depth map) still images.

As mentioned by Besl & Jain [1], the problem is similar to that of object recognition in 2D images, except that it is well posed as the affine variances are lifted from the quasi-3D image. The usual challenges of 2D image recognition, however, such as segmentation and occlusion still apply to 2.5D/3D image recognition.

The context of our study is the recognition of machined parts in the manufacturing industry, for example, for robot control and automated verification of dimensions. Most machined parts have low textural information over much of the surface which contrasts with natural or biological samples, for which many of the approaches in the literature have been developed. Thus, photometric feature recognition-based techniques [2] will suffer from lack of data. In addition, machined parts do not generally possess many so-called interest points, at which the local surface normal varies rapidly [3]. Even if the parts contain interest points, most of them are similar (such as corners of a cuboid or edges of a cylinder), requiring a robust, efficient and powerful classifier as opposed to the simple classifier available through RANSAC [4,5].

By contrast, the approach proposed in the current paper makes use of all the surface coordinate data within the scene, instead of retaining only that from the high-curvature zones where measurement errors are in any case generally at their highest. For the algorithms to perform effectively in the presence of occlusions, a powerful classifier is still required. To our advantage, machined parts adhere to design specifications, lifting classifier requirements for scale invariance. To accomplish this task, we present a powerful probabilistic classifier based on maximum-likelihood, by defining a probability density function (PDF) for the pose of the object, given detected scene features with multiple correspondences to model features. The current paper provides a significantly expanded account of the algorithm, its validation and some applications with respect to a conference publication by the same authors [6].

The organization of the paper is as follows. In §2, we provide a summary of the current literature and trends in 3D object recognition and localization. The novel maximum-likelihood 3D localization is presented in §3 in terms of a probabilistic matching for a general set of features. In §4, we present segmented surfaces as rich features and show how to extract such surfaces from a depth image. We also introduce the form of the PDFs to be used with such surface features. In §5, a gold standard model is created from multiple poses of the object of interest annotated with photogrammetric markers. Using this gold-standard model, we present results in §6 of object localization for both the object on its own and in the presence of clutter. A short comparison of the performance of previously published algorithms on the same datasets is given in §7, before we draw conclusions in §8.

## 2. Summary of existing approaches

As set out by Besl & Jain [1], object recognition involves two integral steps. The first step is to build a labelled model given a collection of non-deformed labelled solid objects (this includes CAD models). The second step can be divided into recognition and localization which are not necessarily achieved in a single step [7]. Throughout the existing literature, there is a clear pattern of steps in object recognition and localization:

(i) feature extraction,
(ii) feature description,
(iii) classification or coarse localization, and
(iv) localization refinement.

### 2.1. Feature extraction

The earliest attempts at object recognition used generalized cylinders, geons and super quadrics [7]. At the time, it was difficult to extract these high-level 3D primitives from 2D or range images, due to the requirement to identify the primitives manually. One significant advantage is that they provide a particularly compact representation [7]. The most commonly used next level of abstraction was that of edge features such as curves [8,9]. These can be extracted from range images but require mechanisms to group compatible features together. This was achieved in a successful object recognition system named 3DPO [8]. The disadvantage of this system was that it was very time inefficient, due to the large number of features present.

The next level of features contain localized points, mainly based on curvature, and allowed 2D object recognition algorithms such as SIFT [10] to be employed. It is interesting to note that edges and high-level 3D primitives have been mainly used in machined part recognition applications, whereas local feature points were used primarily in natural object recognition and registration.

### 2.2. Feature description

Once features have been selected, they need to be represented in such a way that they can be distinguished from other similar features, yet recognized as the same feature when parts of it are missing, for example, due to occlusion [11]. For the high-level 3D primitives, the descriptor involves its parameters and is self-explanatory. However, for generalized curved features, this is not as straightforward or well defined. In the literature, curves are generally described as quadric splines.

For interest feature points there is a multitude of descriptors, mainly categorized under histograms or signatures [10]. Histograms provide less descriptive power, i.e. the ability to discriminate between features, resulting in more false positives, while signatures provide more descriptive power, resulting in false negatives. Some authors have combined the two recently to produce innovative neighbourhood descriptors with balanced descriptive power [11].

### 2.3. Coarse localization

Once correspondences are made between the model and the scene by matching features with descriptors, a classification algorithm is required to identify correspondence groups related to objects that need to be identified. The earliest attempts were to build interpretation trees where each correspondence creates new branches in the tree [12]. When incorporating more correspondences into the tree, some branches of the tree can be pruned so that the tree obeys the correspondence grouping rules [7].

Geometric hashing was another way of representing possibilities so that a pair/tuple of features are selected to determine a unique pose and voted on a hash table for poses. The poses with a large number of votes were selected as candidate poses. The performance of geometric hashing is worst when the object does not exist in the scene, making it inappropriate for recognition [13].

The Hough transform is another voting scheme where each feature votes for candidate poses in pose space known as ‘Hough space’ [14]. The main disadvantages of the Hough transform are (i) the requirement for this sparse Hough space and (ii) the inefficiency of voting for all possible poses, particularly for 6 d.f. problems. Nevertheless, it is one of the most reliable techniques for recognition and localization [15].

RANSAC improves on the geometric hashing by eliminating the voting and by checking for consistency on the candidate position, as determined by a pair/tuple of random features, with a large number of other features [4,5]. In the absence of the sought object, RANSAC is not guaranteed to terminate [4]. This is nevertheless considered a simple classifier and is used widely in almost all interest point-based algorithms.

### 2.4. Fine localization

The last step after coarse localization is to obtain a fine localization, which improves on the accuracy given by the coarse results. Almost always nowadays this is accomplished by an iterative closest point (ICP) algorithm [16,17]. ICP takes all individual points into account to determine the rigid body transformation that minimizes the total distance between closest points in the model and the scene point clouds. ICP is inherently a computationally expensive algorithm that requires a close approximation as the initial pose estimate.

## 3. Maximum-likelihood three-dimensional localizer

From the discussion in §2, it is clear that there is a need for a computationally efficient algorithm that converges and terminates, even when the sought object is absent from the scene, and without large memory requirements. The generalized probabilistic technique of maximum-likelihood estimation presented here offers all these features. In addition, the same algorithm works for both coarse and fine localizations, thus eliminating the need for a separate ICP-type algorithm.

Maximum-likelihood estimation has been used previously to match 2D images, mostly for the classification of image content. Revow *et al.* [18] has used a maximum-likelihood formulation with Gaussian mixture models to detect handwritten digits, which matches models of digits to individual pixels to maximize the likelihood of the model digits. Olson [19] and Greenspan *et al.* [20] have used the method to match objects pairwise by extracting feature regions and matching them with Gaussian mixture models.

The localization problem is essentially one of estimating the pose of an object, given a set of features detected in a scene. In recognizing an object with *M* known features ***m***={***m***_0_,***m***_1_,…,***m***_*M*_} in a scene containing *N* detected features ***s***={***s***_0_,***s***_1_,…,***s***_*N*_}, the pose of the object present in the scene can be estimated by maximizing the likelihood of the pose given the scene features and the known model features. Each of the ***m***_*j*_ and ***s***_*i*_ are in general vector quantities containing as a minimum the coordinates of a point on the feature, with possible additional components to represent information such as curvature, texture etc. In the case of labelled targets used in photogrammetry, one component of these vectors would be the label code.

### 3.1. General formulation

The pose ***Θ*** of an object is in general a 6-component vector (*x*,*y*,*z*,*ω*,*ϕ*,*κ*)^T^ with three translational components (*x*,*y*,*z*) and three Euler angle parameters (*ω*,*ϕ*,*κ*). However, when the object is invariant under translation along, or rotation about, a coordinate axis, the pose may have less than six components. For example, a sphere’s pose is given by only three translational components, whereas that of a cylinder is given by three translational and two angular components. When there are symmetries, multiple equivalent poses also exist. In general terms, the likelihood of a parameter vector, given data, is proportional to the probability of the data, given the parameter [21]. When applied to the current problem, the datum is the scene vector ***s*** and the parameter vector is ***Θ***. Thus, the likelihood of the object being present in a scene with pose ***Θ***, given measured features ***s***, is directly proportional to the probability of observing features ***s*** given the pose of the model ***Θ***:

3.1 L(Θ|s)=P(s|Θ).

If we assume that all the features are independent of one another, the probability of observing all the scene features ***s*** is given by

3.2 $$P(\text{s}|\Theta )\propto \prod_{i=1}^{N}f_{i}({\text{s}}_{i}|\Theta ),$$

where *f*_*i*_(***s***_*i*_|***Θ***) is the PDF for the *i*th feature in the scene, following rotation and translation of the model features by the pose vector.

It is often not known which model feature matches which scene feature. In the most general case, the *i*th scene feature could be matched to any one of the *M* model features, though with differing probabilities depending on how well certain characteristic features of a specific scene/model feature pair match one another. This situation can be represented by writing *f*_*i*_ as a sum of the PDFs *g*_*ij*_ over all the model features as follows:

3.3 $$f_{i}({\text{s}}_{i}|\Theta )=\frac{1}{M}\sum_{j=1}^{M}g_{ij}({\text{m}}_{j}|\Theta )+g_{0}.$$

Here, *g*_*ij*_ is the PDF that specifies the probability of finding the *i*th scene feature at a given location within the scene, due to the *j*th feature of the model after translation and rotation by the pose vector. *g*_0_ is a constant background PDF that deals with the case of no match between the *i*th scene feature and any of the model features. Such a situation can easily arise in a cluttered scene if a feature is found in the scene from somewhere not belonging to the model, and would result in *P*(***s***|***Θ***) becoming equal to zero for all choices of the pose vector.

*g*_*ij*_ can in turn be expressed as a product of PDFs *h*_*ijk*_ for the *O* components *m*_*jk*_ of ***m***_*j*_, assuming that these are independent random variables, as follows:

3.4 $$g_{ij}({\text{m}}_{j}|\Theta )=\prod_{k=1}^{O}h_{ijk}(m_{jk}|\Theta ).$$

When the components of ***m***_*j*_ all come from normal distributions, one can write

3.5 $$h_{ijk}(m_{jk}|\Theta )=\frac{1}{\sigma_{ijk}\sqrt{2\pi }}\exp\left(-\frac{{\left(m_{jk}-s_{ik}\right)}^{2}}{2\sigma_{ijk}^{2}}\right),$$

where *σ*_*ijk*_ is the standard deviation of the distribution.

As previously stated, the feature vectors will normally have one or more components specifying positional information and may have additional components that specify one or more characteristic parameters describing the feature. The characteristic parameters should be chosen to be invariant under rotation and translation, for example, surface curvature. Ideally, these characteristic parameters should also be largely immune to the effect of partial occlusions. The above theoretical derivation, which followed from a direct application of the principle of maximum-likelihood estimation, involves rotation and translation of the model to the scene. However, in many cases the model will be a scanned version of an equivalent component to that appearing in the scene. In such cases, a ‘scene to model’ matching can be used as an alternative to the ‘model to scene’ approach used here.

The computational complexity of the main part of the algorithm follows from (3.2)–(3.5), from which it is clear that *O*(*MNO*) operations are needed for each iteration of the minimization function. This neglects the pre-processing of the data to identify the features, which is generally short by comparison.

### 3.2. An illustration of the algorithm in 2 d.f.

**Figure 1.**
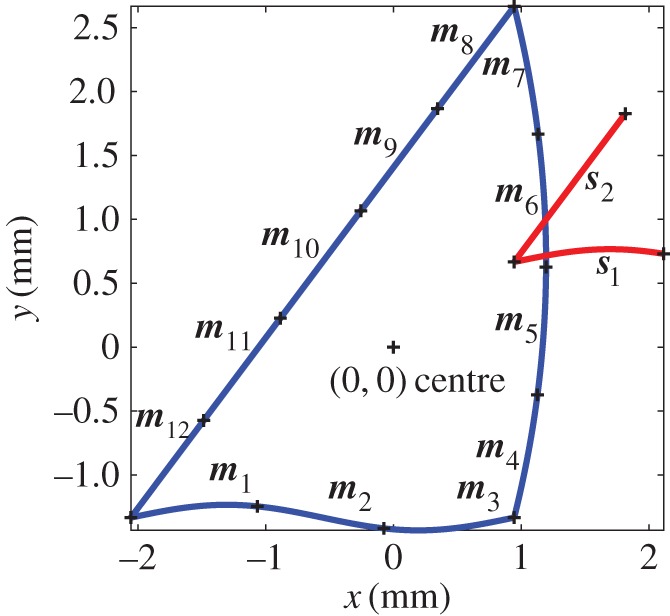
Illustration of the 2 d.f. problem of finding the centre of a triangular model (blue) within the scene (red).

An illustration of the maximum-likelihood approach is given by the curved triangle shown in figure 1. It will be assumed here that the orientation of the triangle (i.e. rotation angle about the axis normal to the *x*-*y* plane) is fixed in the scene to make the problem essentially 2 d.f. Hence, the sought pose vector is ***Θ***=(*x*_c_,*y*_c_)^T^ where *x*_c_ and *y*_c_ are the coordinates of the centroid of the triangle in the scene.

To accommodate the curved nature of the line segments, each segment is divided into several sub-segments. Each sub-segment will be considered as one of the features of the method described in the previous section. The sub-segments are considered to be short enough to be approximated as straight lines, the characteristics of which are the gradient (or angle made with the *x*-axis) and the intercept (or a point on the line). These sub-segments will be referred to as line segments from now on.

The *i*th scene vector can thus be written as ***s***_*i*_=(*x*_*s*_*i*__,*y*_*s*_*i*__,*φ*_*s*_*i*__)^T^, where *x*_*s*_*i*__ and *y*_*s*_*i*__ are the coordinates of the centre of the *i*th scene line segment. *φ*_*s*_*i*__, the angle of the segment with respect to the positive *x*-axis, is chosen as the characteristic parameter of a line segment as it is invariant under translation of the model. Similarly, the model vector after translation by ***Θ*** may be written ***m***_*j*_=(*x*_*m*_*j*__+*x*_c_, *y*_*m*_*j*__+*y*_c_, *φ*_*m*_*j*__)^T^, where *x*_*m*_*j*__ and *y*_*m*_*j*__ are the coordinates of the centre of the *j*th model line segment relative to the centroid of the model triangle and *φ*_*m*_*j*__ is the angle of the segment with respect to the positive *x*-axis.

The PDF *h*_*ijk*_(*m*_*jk*_|***Θ***) describing the probability of a match between two line segments on account of their position and the characteristic parameter can be expressed in its simplest form using (3.5). When assigning appropriate values for the *σ*_*ijk*_, however, it is important to recognize that there is significantly greater uncertainty in the position of the line in the direction parallel to the segment than there is in the normal direction. This is because the locations of the edges of the line segments may be chosen arbitrarily when the data contain occlusions, whereas the uncertainty in the normal direction is dictated by the accuracy of the measuring system and the variation of the curve segment in the normal direction.

It is, therefore, more appropriate to work with the transformed variables $m_{jk}^{\prime }$ and $s_{ik}^{\prime }$ as follows:

3.6 $$\begin{array}{rl} \left(\begin{array}{c} m_{j1}^{\prime }\\ m_{j2}^{\prime } \end{array}\right) & ={\text{R}}_{m_{j}}\left(\begin{array}{c} m_{j1}\\ m_{j2} \end{array}\right)\\ \;\text{and}\;\left(\begin{array}{c} s_{i1}^{\prime }\\ s_{i2}^{\prime } \end{array}\right) & ={\text{R}}_{m_{j}}\left(\begin{array}{c} s_{i1}\\ s_{i2} \end{array}\right) \end{array}\},$$

where ***R***_*m*_*j*__ is the rotation matrix associated with the *j*th model line segment.

3.7 $${\text{R}}_{m_{j}}=\left(\begin{array}{cc} \cos m_{j3} & \sin m_{j3}\\ -\sin m_{j3} & \cos m_{j3} \end{array}\right).$$

When applying (3.5), the variables $m_{jk}^{\prime }$ and $s_{ik}^{\prime }$ are used in place of *m*_*jk*_ and *s*_*ik*_, for the cases *k*=1,2. Then $m_{jk}^{\prime }-s_{ik}^{\prime }$ represent the separation between the scene and model segment locations measured in the parallel (*k*=1) and normal directions (*k*=2) to the model line segment. The corresponding uncertainties are $\sigma_{ijk}^{\prime }$: $\sigma_{ij1}^{\prime }$ should be chosen to be comparable in value to the line segment length, whereas $\sigma_{ij2}^{\prime }$ should be chosen considering the measurement uncertainty of the measuring instrument in the normal direction and the variation of the curve in the normal direction.

**Figure 2.**
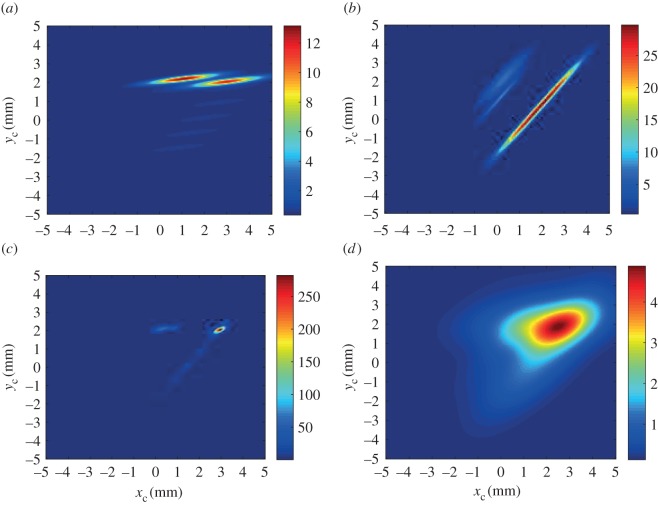
The PDFs *f*_1_(*s*_1_) (*a*) and *f*_2_(*s*_2_) (*b*) corresponding to the line segments *s*_1_ and *s*_2_ in the scene of figure 1, as a function of the pose vector components *x*_c_ and *y*_c_. (*c*) Likelihood function *f*_1_(*s*_1_)*f*_2_(*s*_2_) given by (3.2). (*d*) Same as (*c*) but with broadened PDFs (see text for details).

**Figure 3.**
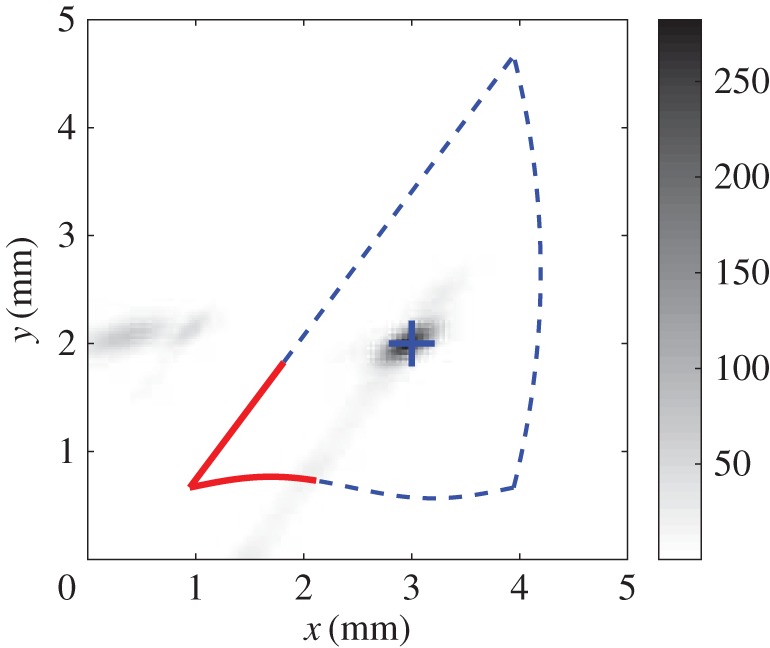
The model shifted to the most likely point in the scene. The likelihood function from figure 2*c* is superimposed together with the maximum-likelihood estimate (+) for the model position in the scene.

Figure 2*a* and *b* illustrate the PDFs *f*_1_(***s***_1_) and *f*_2_(***s***_2_), respectively, where ***s***_1_ and ***s***_2_ are the two scene line segments shown in figure 1. The parameters chosen for this example were $\sigma_{ij1}^{\prime }=0.96$ mm, $\sigma_{ij2}^{\prime }=0.1$ mm, $\sigma_{ij3}^{\prime }=0.3$ rad and $g_{0}=(1/24\sqrt{2})\;{\mathrm{mm}}^{-2}\;{\mathrm{rad}}^{-1}$. When both of these are combined to form *P*(***s***|***Θ***) as shown in figure 2*c*, the resultant peak location is seen to become close to the actual centre (*x*_c_,*y*_c_)^T^=(3,2)^T^ mm, despite the presence of a large number of features in the model that have no corresponding features in the scene to match to. The effect of artificially broadening the distributions by increasing $\sigma_{ij1}^{\prime }$ and $\sigma_{ij2}^{\prime }$ by factors of 2 and 8, respectively, is seen in figure 2*d*. Once again the peak in *P*(***s***|***Θ***) is seen to lie close to (3,2)^T^ mm. In the following sections, the use of a small baseline probability and broad dispersions allows an initial estimate of the model location to be obtained. Narrowing the dispersions then narrows the peak, thus improving the accuracy in the presence of noise. Figure 3 shows the result of translating the scene to the model using the maximum-likelihood estimator for the pose vector. This simple example illustrates the basis for the combined global optimization and local refinement stages offered by the maximum-likelihood technique, which contrasts with other approaches from the literature in which a different algorithm is required for each.

## 4. Surface features

In machined parts where large smooth surfaces are more common than interest points with high curvatures, interest point feature-based object recognition loses information present in the surfaces. In our approach, smooth surface patches are considered as features constraining the pose of the object. Similar feature classification is conducted in [22], in order to segment unknown 3D objects. However, unlike in [22], surface segmentation is based on surface normals as we have a high-accuracy dense point cloud at our disposal. In this paper, we will be considering only surfaces, which are considered least in the contemporary literature.

### 4.1. Segmentation of surfaces

Surfaces from a 3D depth map are segmented on the basis of surface normal directions. The depth map provides (*x*,*y*,*z*) coordinates for each image pixel, the location of which in the image sensor array is defined by indices (*p*,*q*). A separate 1-bit image provided by the scanner indicates which pixels contain valid data and which are to be disregarded. To find the surface normals both reliably and efficiently, an *N*_*p*_×*N*_*p*_ neighbourhood centred on (*p*,*q*) is selected, where *N*_*p*_ took the value 15 for the results reported here. If all pixels in the neighbourhood are valid, an orthogonal polynomial decomposition is performed [23] to determine the separable quadratic surface on (*p*,*q*). If not, a quadratic surface fit is performed using singular value decomposition (SVD). Once the quadratic surface coefficients on (*p*,*q*) are found, one can define the surface normal from the first-order coefficients and the Gaussian (H) and mean (K) curvatures from the first- and second-order coefficients.

The next step is to threshold the image based on H and K in order to remove points with high curvature. This removal essentially separates surfaces into segments. Owing to the high noise typically present in the H and K maps, however, not all high-curvature points are removed and a few may remain that connect some surface segments.

**Figure 4.**
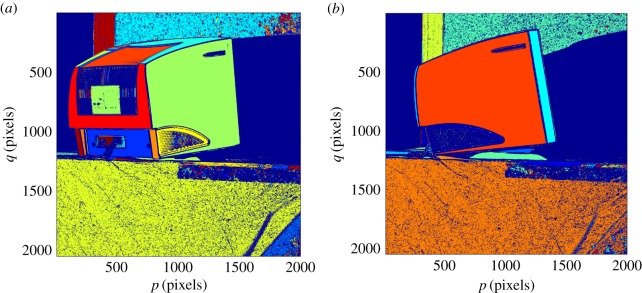
Surface segmentation of shape data from two poses (*a*,*b*) of a CRT monitor. Different segments are shown in different colours.

A connected component analysis based on surface normals and 3D position is then performed. This allows neighbouring surface normals to vary by up to a maximum defined angle (0.05 *rad* in the current study) within a connected surface segment. The result of surface segmentation of an illustrative part is shown in figure 4 for two different poses (or views). The 3D data used throughout the paper were acquired by a *Phase Vision Quartz 1200 DBE* scanner which uses white light projected fringes, processed using the ‘reversed exponential’ method from [24], to provide up to 4×10^6^ independent coordinates.

### 4.2. Probability density function of a surface

Once the surface has been segmented as described in the previous subsection, the detected surfaces are cut into smaller patches if either the surface area is too large, or the surface normal variation is too large to be captured by a single quadratic surface. This process is analogous to that of dividing line segments into sub-segments in the 2 d.f. example considered in §3b. As mentioned previously, the rationale behind considering several quadratic patches is to allow for some degree of occlusions in the scene. In the presence of occlusions, the patch separation basis will not be the same even for the same surface. Hence, one needs to allow for such differences by appropriate choice of the variances of the PDF describing the surface patches, as will be discussed later.

**Figure 5.**
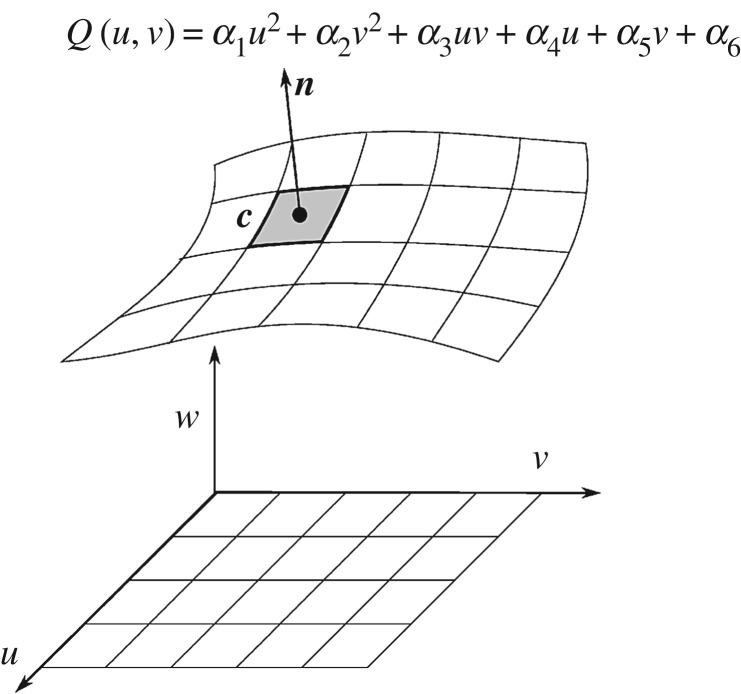
Representation of a surface as a collection of surface patches and properties of one of those patches.

Figure 5 illustrates the separation of a large surface into smaller patches. The principal axes of a surface (*u*−*v*−*w*) are first determined using a principal component analysis (PCA) on the point cloud of the surface. The surface is cut parallel to *u*−*v* axes as square domains in *u*−*v* as shown in figure 5.

A second PCA is then performed on the point cloud of each surface patch, allowing the patch to be represented by a set of quadratic coefficients *α*_*l*_ (*l*=1,2,…,6) defining the quadric surface *Q*(*u*,*v*)=*α*_1_*u*^2^+*α*_2_*v*^2^+*α*_3_*uv*+*α*_4_*u*+*α*_5_*v*+*α*_6_, a surface normal ***n*** and a centre coordinate ***c***. Note that ***c*** is the origin of the (u-v) domain, while ***n*** is the *w* axis of the (*u*−*v*−*w*) frame. Each patch is then considered as an independent feature entity. During this study, we did not consider any characteristic parameters to match patches, even though one could use quadratic coefficients, average mean or Gaussian curvature, etc. as characteristic parameters. Surface normal information is used, by analogy, with the line segment angles used in the 2 d.f. example from Section 3b. There is, however, a distinction between the 2 d.f. and 6 d.f. cases: in the former, the segment angle is a true characteristic feature in that it is invariant under application of the pose vector, whereas in the latter it is not. Nevertheless, the surface normal can usefully be used in the matching and is represented here by the third component of the model/scene vectors. All patches in the model are therefore considered as potential matches to all the patches in the scene.

**Figure 6.**
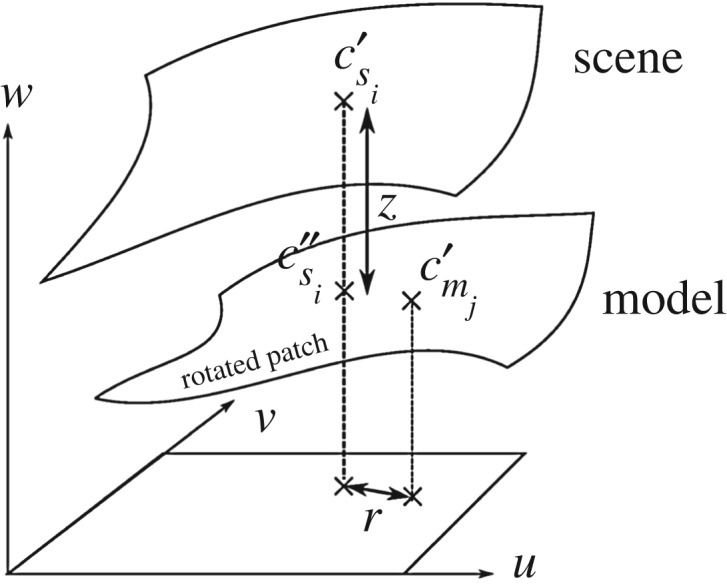
Definition of vertical and radial distances between two surfaces.

The probability of patch *i* in the scene matching patch *j* in the model is calculated from the vertical and radial distances between the surfaces, and the angle between the surface normals, as shown in figure 6. As noted in the illustration, ***m***_*j*_ is the *j*th model feature vector transformed by the 6 d.f. pose estimate ***Θ***; thus, the centre ***c***_*m*_*j*__ , the normal ***n***_*m*_*j*__ , the (*u*−*v*−*w*)_*m*_*j*__ frame, the quadric surface *Q*_*m*_*j*__, the rotation matrix ***R***_*m*_*j*__ and the translation vector ***T***_*m*_*j*__ relating the scene frame to the (*u*−*v*−*w*)_*m*_*j*__ frame have all been transformed by ***Θ***.

The centres and the surface normals of the two patches are transformed onto the model patch’s *u*−*v*−*w* frame:

4.1 $$\begin{array}{rl} {\text{C}}_{s_{i}}^{\prime } & ={\text{R}}_{m_{j}}{\text{C}}_{s_{i}}+{\text{T}}_{m_{j}}\\ {\text{C}}_{m_{j}}^{\prime } & ={\text{R}}_{m_{j}}{\text{C}}_{m_{j}}+{\text{T}}_{m_{j}}\\ \;\text{and}\;{\text{n}}_{s_{i}}^{\prime } & ={\text{R}}_{m_{j}}{\text{n}}_{s_{i}} \end{array}\},$$

where ${\text{ c}}_{s_{i}}^{\prime }=(c_{s_{i}}^{\prime (u)},c_{s_{i}}^{\prime (v)},c_{s_{i}}^{\prime (w)})$, ${\text{ c}}_{m_{j}}^{\prime }=(c_{m_{j}}^{\prime (u)},c_{m_{j}}^{\prime (v)},c_{m_{j}}^{\prime (w)})$ and ${\text{ n}}_{s_{i}}^{\prime }=(n_{s_{i}}^{\prime (u)},n_{s_{i}}^{\prime (v)},n_{s_{i}}^{\prime (w)})$. The radial distance *r* between the patches is taken as the distance between the projections onto the *u*−*v* plane. The vertical distance *z* between the patches is taken as the distance along the straight line segment parallel to the *w*-axis of the model patch that passes through the scene patch centre as shown in figure 6. The intersection between the model patch and the above-mentioned straight line is given by

4.2 $$c_{s_{i}}^{\prime \prime (w)}=Q_{m_{j}}(c_{s_{i}}^{\prime (u)},c_{s_{i}}^{\prime (v)}).$$

Hence, the vertical (*k*=1) and radial (*k*=2) separations *m*_*jk*_−*s*_*ik*_ between the patches are given by

4.3 $$\begin{array}{rl} m_{j1}-s_{i1} & =z=c_{s_{i}}^{\prime \prime (w)}-c_{s_{i}}^{\prime (w)}\\ \;\text{and}\;m_{j2}-s_{i2} & =r=\sqrt{{\left(c_{m_{j}}^{\prime (u)}-c_{s_{i}}^{\prime (u)}\right)}^{2}+{\left(c_{m_{j}}^{\prime (v)}-c_{s_{i}}^{\prime (v)}\right)}^{2}} \end{array}\}.$$

The model surface normal varies on the model patch and should similarly be estimated at ${\text{ c}}_{s_{i}}^{\prime \prime }$ using *Q*_*m*_*j*__.

4.4 $${\text{n}}_{s_{i}}^{\prime \prime }=\frac{\left(-\partial Q_{m_{j}}/\partial u{|}_{\left(c_{s_{i}}^{\prime (u)},c_{s_{i}}^{\prime (v)}\right)},-\partial Q_{m_{j}}/\partial v{|}_{\left(c_{s_{i}}^{\prime (u)},c_{s_{i}}^{\prime (v)}\right)},1\right)}{\sqrt{1+{\left(\partial Q_{m_{j}}/\partial u{|}_{\left(c_{s_{i}}^{\prime (u)},c_{s_{i}}^{\prime (v)}\right)}\right)}^{2}+{\left(\partial Q_{m_{j}}/\partial v{|}_{\left(c_{s_{i}}^{\prime (u)},c_{s_{i}}^{\prime (v)}\right)}\right)}^{2}}}.$$

The difference in the surface normals *m*_*j*3_–*s*_*i*3_ is defined as the angle between ${\text{ n}}_{s_{i}}^{\prime }$ and ${\text{ n}}_{s_{i}}^{\prime \prime }$ , and $\sigma_{ij1}^{\prime }$, $\sigma_{ij2}^{\prime }$ and $\sigma_{ij3}^{\prime }$ are the standard deviations in vertical distance, radial distance and surface normal, respectively. The position of the boundaries between patches is arbitrary and therefore the radial standard deviation $\sigma_{ij2}^{\prime }$ is chosen to be comparable to the patch domain dimensions. By contrast, the vertical standard deviation $\sigma_{ij1}^{\prime }$, controlled by the measurement accuracy of the instrument and the patch variation, was chosen here to be the standard deviation of the *w* variation of the patch points. Lastly, the angle standard deviation $\sigma_{ij3}^{\prime }$ was calculated from the variance of the surface normals within the patch. For instance, planar patches would have narrow variances in angle and vertical distance, whereas more curved patches have broader variances in the same.

## 5. Building a gold standard model

The first step in the object recognition process is to create a model of a non-deformed object that is to be sought within the scene. This section presents the method used to build such a ‘gold standard’ model by employing some of the techniques discussed above.

### 5.1. Method

To build a gold standard model, the object of interest was first annotated by circular markers. Point clouds from multiple views of the object were obtained, with some overlap between views. Through the use of a Hough transform of the texture images in the neighbourhood of the markers, the marker ellipse centres were determined to sub-pixel accuracy. By matching the corresponding ellipses across views, and using singular value decomposition to obtain the rigid transformation, the poses of all the views were obtained relative to one view.

Each view was then segmented as described in §4a. At this point, one is left with multiple point clouds each defined in their own (*p*,*q*) domain, the pose of which is known. For each point in a given view, the closest points in all other views are found. A 10 mm threshold was used to filter out ill-matching points. This gives an initial connectivity between surfaces of different views. Owing to noise, close multiple surfaces in one view may present as one bigger surface in another view. This is quite common when the surface points are close to the extremes of the field of view. In the current study, the larger surface has been retained in such cases instead of keeping multiple smaller surfaces.

### 5.2. Background points/surface removal

Once the surface segmentation of the gold standard model is complete, some surfaces may exist in the gold standard model that belong to the background, such as the platform the object is placed upon. These should be removed from the point cloud before merging into a gold standard model.

To identify points not belonging to the part at an early stage, one can remove points without any correspondences in another view. This, however, requires one to capture the part so that each surface is overlapped completely in another view. A better technique is to make use of the surface segmentation information as well. If a segmented surface contains a majority of points that do not have closest point correspondences, then that surface can be removed from the gold standard model. In addition, one would only want to keep surfaces large enough to represent the major features of the part.

### 5.3. The model of the part

Once a correspondence graph is built between surfaces in all views, all points in all the views belonging to the same surface are used to build the parameters of that surface of the gold standard model.

**Figure 7.**
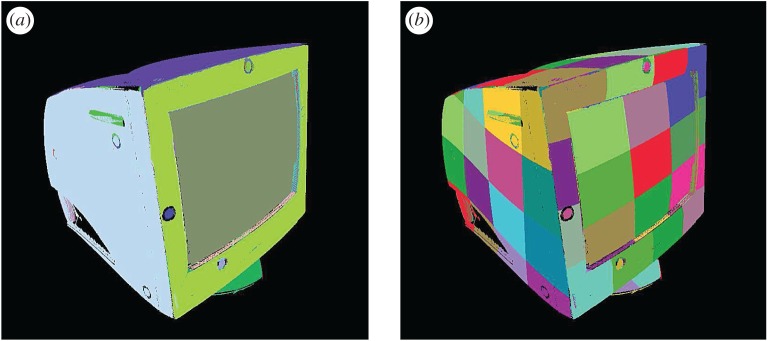
The gold standard model showing (*a*) segmented surfaces and (*b*) surfaces cut into smaller patches as features.

The gold standard model derived using the above techniques is shown in figure 7 for the case of a computer cathode ray tube (CRT) monitor. In the part shown, nine major surfaces were identified which were represented by partitioning into sub-segments.

The gold standard model contains all the points related to the recognized surfaces. Some internal surface segments are seen to be missing. This is due to the fact that they do not form a connected surface and have too many high-curvature points. Such regions with high interest point density could potentially be used for a more complete and robust implementation. They are ignored for now, however, in view of the hypothesis underlying this paper, namely that surfaces without interest points also contain enough information for successful object recognition and localization using the maximum-likelihood algorithm.

## 6. Results

In this section, the results of identifying a part in an arbitrary pose in isolation, followed by the more challenging problem of identification of a part in a cluttered scene, are presented.

### 6.1. Pose estimation in isolation

As mentioned previously, the PDF was formed by taking the product over the individual PDFs for all model surface patches. The PDF of each model surface patch is the sum of PDFs of that surface patch matching all scene surface patches with the addition of the baseline probability for the non-matching condition. The natural logarithm of the combined PDF was taken as the logarithm of the likelihood (i.e. log likelihood) function. The objective of pose estimation is to find the maximum of the log-likelihood function in six-dimensional pose space.

The optimization algorithm starts with an initial pose vector of (0,0,0,0,0,0) with constraint limits for *x*,*y*,*z* of 3 m (the range of the structured light scanner used) and for angular parameters *ω*, *φ* and *κ* of ±*π*, ±*π*/2 and ±*π*, respectively. The optimization algorithm moves along the local steepest gradient of the log-likelihood function. MATLAB function fmincon was used for this purpose.

The main obstacle to using this optimization algorithm is that the log-likelihood function contains narrow peaks with a plateau region over much of the rest of the parameter space. If the initial pose estimate is on such a plateau, the algorithm is unable to find a non-null gradient, resulting in premature termination. To avoid this, the log-likelihood function was broadened by scaling the standard deviations $\sigma_{ij1}^{\prime }$, $\sigma_{ij2}^{\prime }$ and $\sigma_{ij3}^{\prime }$ of the model surface patches until the algorithm is able to move away from the initial pose estimate. For the part used in the study, the average value of standard deviations were found to be $\sigma_{ij1}^{\prime }=1$ mm, $\sigma_{ij2}^{\prime }=15$ mm and $\sigma_{ij3}^{\prime }=0.01$ rad. The algorithm could move freely towards a maximum when $\sigma_{ij2}^{\prime }$ was scaled by a factor of 3, and $\sigma_{ij1}^{\prime }$ and $\sigma_{ij3}^{\prime }$ were scaled by a factor of 27. To achieve global optimization with most matches with the model surface patches, a very low background probability value of *g*_0_=1.37×10^−47^ mm^−2^ rad^−1^ was used. The model created as mentioned in §5 contained 73 patches (*M*=73), while the scene contained 34 patches (*N*=34). It should be pointed out that, in general, the appropriate choice of the $\sigma_{ijk}^{\prime }$ parameters for the fine localization is related to the intrinsic measurement uncertainty of the scanner ($\sigma_{ij1}^{\prime }$, $\sigma_{ij3}^{\prime }$), and to the patch size and complexity of the surface profile of the part being measured ($\sigma_{ij2}^{\prime }$, $\sigma_{ij3}^{\prime }$). The scaling factors for the coarse localization are determined empirically, by increasing their values until the model is able to move significantly.

**Figure 8.**
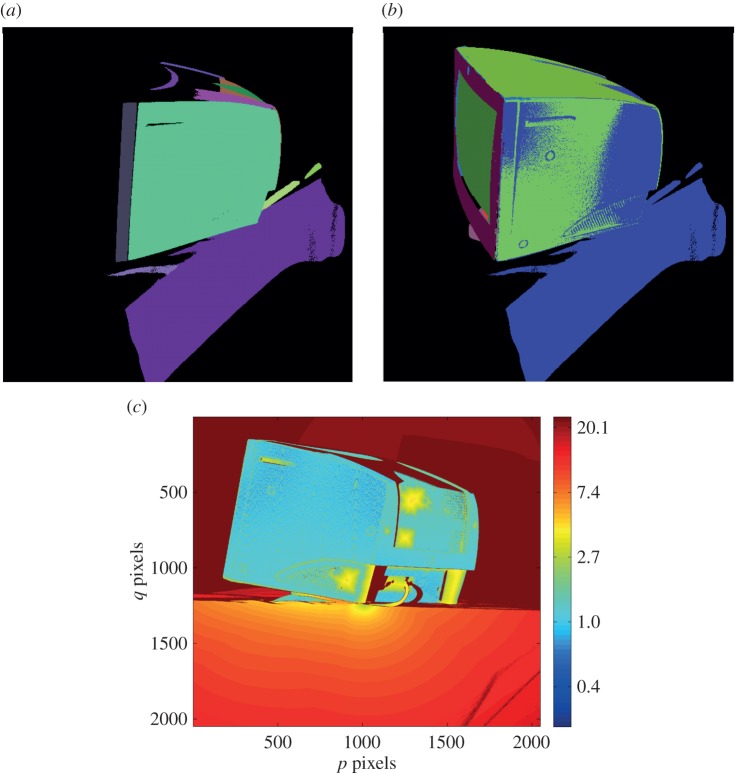
Localization for an isolated object with minimal clutter. (*a*) Colour-coded 3D segmented scene (one colour per surface); (*b*) scene surfaces displayed in one colour (blue) with colour-coded 3D model surfaces in the estimated pose overlaid within the scene; (*c*) logarithmic error map (units: millimetre).

After finding the initial estimate of the pose vector, the optimization algorithm was rerun without the scaling of the standard deviations of the PDF so as to obtain a more accurate estimate of the pose vector. Figure 8*a* shows the segmented scene and figure 8*b* shows the scene once the model part has been recognized and placed within the scene. The match between the part locations in (*a*) and (*b*) shows that reasonably accurate pose estimation of the part in isolation has been achieved. A slight misalignment in one of the side walls of the part is nevertheless visible in figure 8*b*. To quantify the misalignment, figure 8*c* shows the alignment error of each point in the scene from the corresponding closest point in the model, calculated using a standard nearest neighbour-based closest point algorithm.

From figure 8*c*, one can see that the alignment error of the recognized part is only in the range of 0.5–1.5 mm. This can be regarded as a good achievement considering that only surface segments are used for localization. It is likely that one can improve on this value, and make the algorithm even more robust, by including in the algorithm point and line features in addition to the surface features employed here.

### 6.2. Recognition and pose estimation in clutter

The performance of the algorithm was tested here with a moderately cluttered scene containing other surfaces that are similar to the surfaces of the part of interest.

The model in this case did not start moving until the standard deviations of surface patches were increased by a factor of 5 for $\sigma_{ij2}^{\prime }$ and by a factor of 125 for $\sigma_{ij1}^{\prime }$ and $\sigma_{ij3}^{\prime }$. A very low background probability value of *g*_0_=1.37×10^−47^ mm^−2^ rad^−1^ was again used to maximize the number of model surface patch matches. The scene in this cluttered case contained 58 patches (*N*=58).

**Figure 9.**
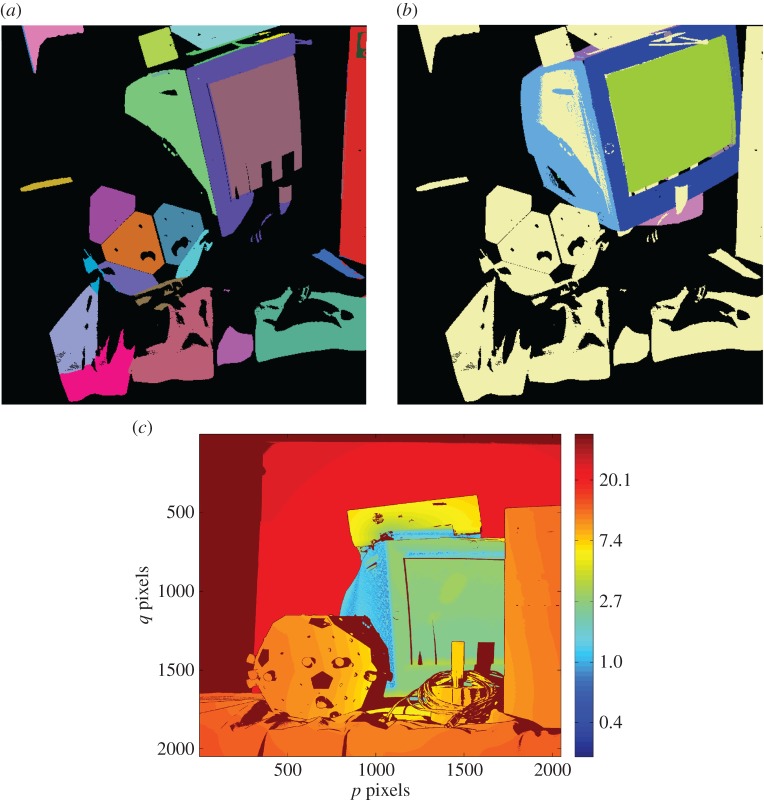
Object localization with moderate clutter. (*a*) 3D segmented scene; (*b*) scene surfaces displayed in one colour (light-yellow) with colour-coded 3D model surfaces in the estimated pose overlaid within the scene; (*c*) logarithmic error map (units: millimetre).

**Figure 10.**
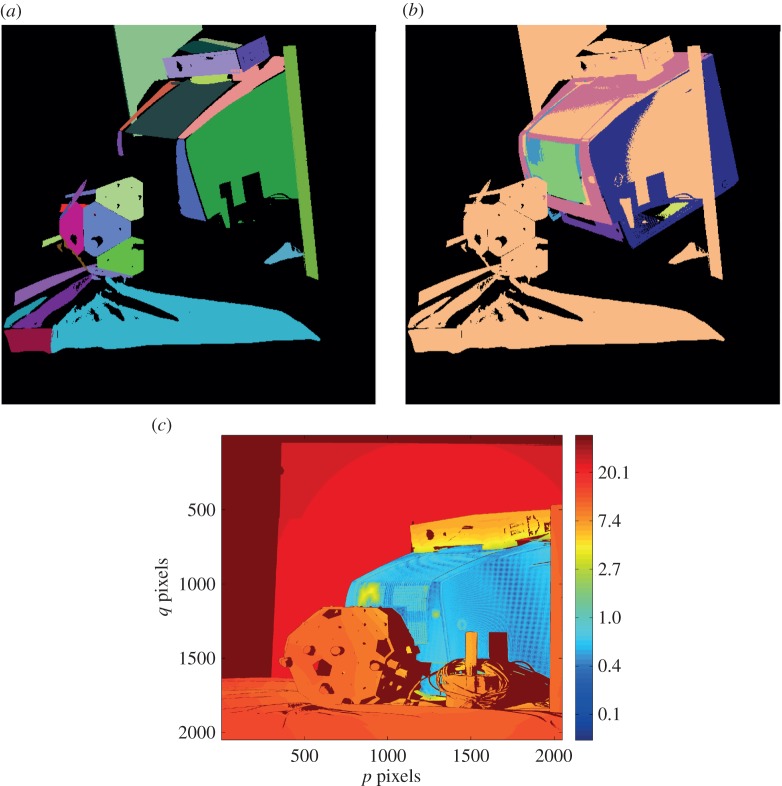
Object localization with moderate clutter. (*a*) 3D segmented scene; (*b*) scene surfaces displayed in one colour (cream) with colour-coded 3D model surfaces in the estimated pose overlaid within the scene; (*c*) logarithmic error map (units: millimetre).

Figure 9*a* shows the segmented cluttered scene in 3D. Figure 9*b* shows the cluttered scene in 3D with the localized model where the segmented surfaces are shown in different colours in the 3D model, while the scene is presented with just a single colour. In the scene, only a handful of high-curvature points are visible but the three mutually orthogonal sides of the part clearly define its pose. Figure 9*c* shows the error map and as is the case with the object localization in isolation, the alignment error of the recognized part is in the range of 0.5–1.5 mm. Another cluttered scene is shown in figure 10*a* with the clutter and the object in different orientations. Figure 10*b* shows the localized model with segmented surfaces in different colours superimposed on the scene shown in a single colour. In this scene, a large area of three mutually orthogonal surfaces are visible from the viewpoint, enabling one to obtain the pose to a higher accuracy. The error map shown in figure 10*c* shows that the alignment error between the object in the scene and the localized model is in the range of 0.2–1.0 mm, with a root mean square error value of 0.3 mm.

These examples illustrate the point that surfaces normally ignored by *interest point algorithms* do contain an abundance of information that can be used by the maximum-likelihood algorithm to accurately estimate object pose, even in the presence of significant clutter.

### 6.3. Objects with more curved surfaces

**Figure 11.**
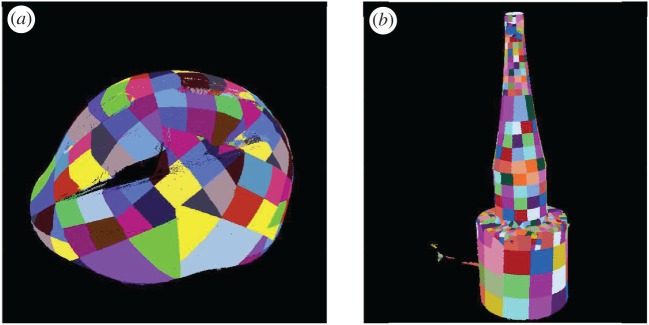
Gold-standard 3D model of (*a*) a bike helmet and (*b*) a conical transducer with an exponential profile.

To provide evidence that the maximum-likelihood method is successful in determining the pose not only of objects with close-to-planar surfaces like the CRT monitor, but also objects with more curved surfaces, the method was applied to two further samples: a bike helmet and a conical transducer with an exponential profile (high-power ultrasonic sonotrode). The gold standard models constructed are shown in figure 11*a* and *b* for the bike helmet and the transducer and had 157 and 444 surface patches, respectively. The average values of standard deviation for the bike helmet patches were $\sigma_{ij1}^{\prime }=0.58$ mm, $\sigma_{ij2}^{\prime }=9.25$ mm and $\sigma_{ij3}^{\prime }=0.19$ rad, whereas those for the transducer were $\sigma_{ij1}^{\prime }=0.23$ mm, $\sigma_{ij2}^{\prime }=4$ mm and $\sigma_{ij3}^{\prime }=0.12$ rad.

**Figure 12.**
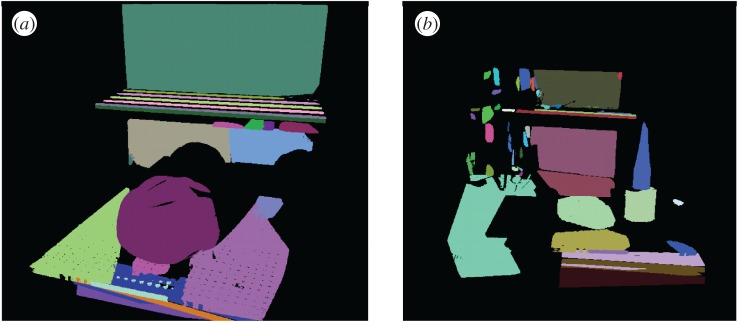
Segmented scenes with (*a*) a bike helmet and (*b*) a conical transducer with an exponential profile.

**Figure 13.**
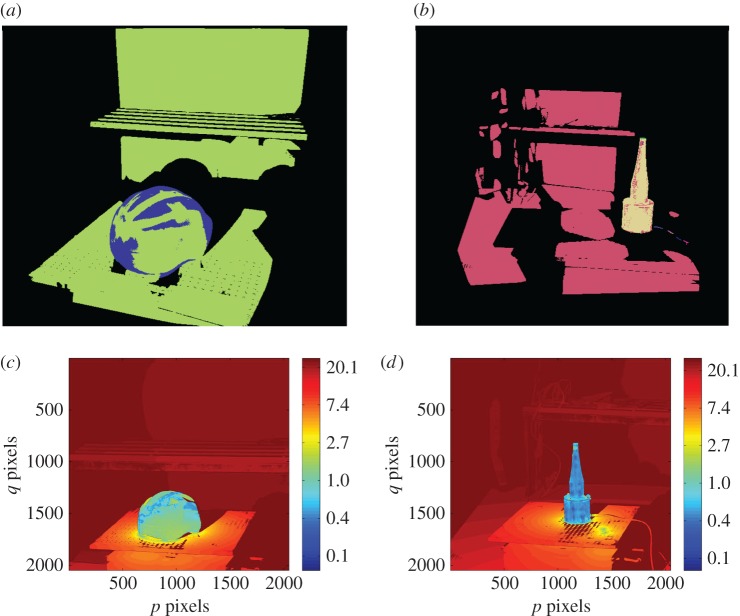
Rotated translated model overlaid within the (*a*) bike helmet scene where the model is in blue, (*b*) transducer scene where the model is in cream. The logarithmic error map (units: millimetre) for (*c*) bike helmet and (*d*) transducer.

Two scenes with each object on its own with a rotary table and little background clutter were used for registration as shown in figure 12*a* and *b*. For the rough estimation, $\sigma_{ij2}^{\prime }$ was scaled by 3, and $\sigma_{ij1}^{\prime }$ and $\sigma_{ij3}^{\prime }$ were scaled by 27 for both models. After the rough estimation, the pose refinement was conducted without scaling factors. The results thus obtained are shown in figure 13*a* and *b* for the bike helmet and the transducer, respectively, and they confirm that the model is aligned to the correct orientation of the object within the scene.

The logarithmic error maps between the scenes and the rotated translated models are shown in figure 13*c* and *d*, respectively. The mean error within the object in the scene is 1.3 mm and 0.26 mm for the bike helmet and the transducer, respectively.

### 6.4. Object recognition

In the previous subsection, it was shown that the maximum-likelihood algorithm can locate an object in a scene in isolation. The same algorithm can, however, also be used for object recognition. Object recognition is the step of identifying whether an object of interest is present in the given scene. When the object localization is halfway completed with the broader PDF, one can find the likelihood value of the rough pose estimate, which is related to the number of closely matching surface patches. The presence or absence of the object can then be decided by applying a threshold to the maximum of the likelihood function, the value of which is dependent on the application.

Table 1 shows the maximum value of the normalized log-likelihood function after the coarse localization step, for matches between each of the three models and three scenes, where each scene contains just one of the objects in isolation. The normalized log-likelihood function is calculated by dividing the sum of log of *f*_*i*_(***s***_*i*_|***Θ***) in equation (3.2) by the total number of scene features *N*. As can be seen, the maximum of the normalized log-likelihood function is generally highest when the corresponding model is present in the scene. The bike helmet model, however, shows a high maximum value of normalized log-likelihood in the presence of the sonotrode. This is due to the relative size and the presence of some surface patches similar to those of the bike helmet in the sonotrode scene.

**Table 1.** The maximum value of the normalized log-likelihood function (i.e. $(1/N)\sum_{i=1}^{N}$ log *f*_*i*_(***s***_*i*_|***Θ***)) calculated for scenes containing each object calculated with each model after the coarse localization step.

model	CRT monitor scene	bike helmet scene	sonotrode scene
CRT monitor	−39.38	−50.19	−63.30
bike helmet	−61.06	−36.60	−39.18
sonotrode	−96.12	−88.39	−43.02

The true-positive and false-positive recognition rates for the three models were calculated over 25 scenes containing the CRT monitor in isolation, and the bike helmet and the sonotrode, both in isolation and in combination, for varying threshold levels of the log-likelihood function. The true-positive rate is defined as the ratio of correct identifications of an object’s presence to object ‘present’ occurrences. The false-positive rate is defined as the ratio of incorrect identifications of an object’s absence to object ‘absence’ occurrences.

**Figure 14.**
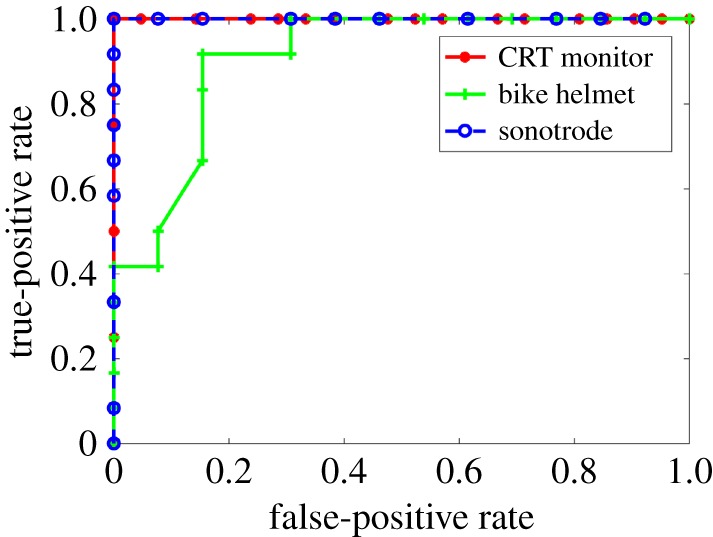
ROC curve for object recognition based on the threshold value for the normalized log-likelihood function.

The resulting receiver operating characteristic (ROC) curves, which display the trade-off between true-positive and false-positive rates as the threshold of the log-likelihood function is varied, are shown in figure 14. For the scenes considered in this study, both the CRT monitor model and the sonotrode model have perfect recall rates for the three objects considered. The bike helmet is the weakest model of the three in that the sonotrode is intermittently recognized as a bike helmet in background clutter. This is consistent with the values presented in table 1. The perfect values obtained for two of the curves may be due to the fact that the differences in sizes of the three objects is significantly larger than the values for standard deviation used in the likelihood function for coarse estimation ($\sigma_{ij1}^{\prime }=6.21$ mm for the sonotrode, $\sigma_{ij1}^{\prime }=15.66$ mm for the bike helmet, $\sigma_{ij1}^{\prime }=27.0$ mm for the CRT monitor). The object size can be characterized in simple terms by the bounding box, which for the CRT monitor is approximately 450×400×450 mm^3^, compared to 400×120×120 mm^3^ for the sonotrode and 250×180×140 mm^3^ for the bike helmet. A further reason for the perfect recall rates may be the rather limited size of the datasets analysed. The results nevertheless demonstrate the potential of this approach to the task of recognizing a modelled object within a 3D scene.

## 7. Comparison

To evaluate the maximum-likelihood algorithm against some of the state-of-the-art feature point-based recognition techniques, results obtained using a standard object recognition pipeline for the CRT monitor are presented in this section.

To successfully recognize an object, the keypoints must first be repeatable across different views of the same section of the object of interest. Secondly, the chosen descriptors of those repeatable keypoints must be equal, where equality is defined by a threshold of the differences between descriptors. Therefore, two studies were conducted—one for repeatability of keypoints and one for repeatability of descriptors—for two types of widely used keypoint types and descriptor types.

### 7.1. Repeatability of keypoints across views

In this section, the repeatability of the Intrinsic Shape Signature (ISS) [25] and Harris 6D [26] detectors, developed as part of the open-source software point cloud library (PCL), was assessed. PCL version 1.7.2 was used for all the analysis presented here. In these algorithms, the surface normal at each point in the point cloud was calculated by fitting a plane to a neighbourhood of points within a sphere of a chosen radius (*R*_norm_) using functions pcl::NormalEstimation::setKSearch and pcl::NormalEstimation::compute. The keypoints were then extracted by comparing each point along with its surface normal to a neighbourhood determined by another radius value (*R*_keypoint_) using functions pcl::ISSKeypoint3D::setSalientRadius for ISS and pcl::HarrisKeypoint6D:: setRadiusSearch for Harris6D detectors. Once the keypoints were extracted, a non-maxima suppression algorithm was run on the extracted keypoints in order to select only the most prominent keypoints within a neighbourhood determined by another radius value (*R*_nonmax_) using functions pcl::ISSKeypoint3D::setNonMaxRadius and pcl::HarrisKeypoint6D::setRadius. In a study conducted on PCL keypoints [27], the authors used 4 times the point cloud resolution (average distance between all closest pairs of points) for *R*_norm_ and *R*_nonmax_, whereas *R*_keypoint_ was varied between 6 and 15 times the cloud resolution. The study in [27] suggested that ISS is more repeatable than the Harris 6D detector, but a different study concluded that there is no such difference [28].

In the current study, the point clouds that were used to create the gold standard were used to estimate repeatability across views, as the relative poses of the views are known beforehand from the photogrammetric markers on the object. To achieve acceptably short run times (a couple of minutes rather than an hour on a desktop PC), the original point clouds were down-sampled by 3 along each axis, thus reducing the dataset sizes by a factor of 9. The resulting cloud resolution was approximately 1.2 mm. *R*_norm_ and *R*_nonmax_ were both chosen to be 5 mm, while *R*_keypoint_ was chosen to be 10 mm in order to closely resemble the study conducted in [27].

**Figure 15.**
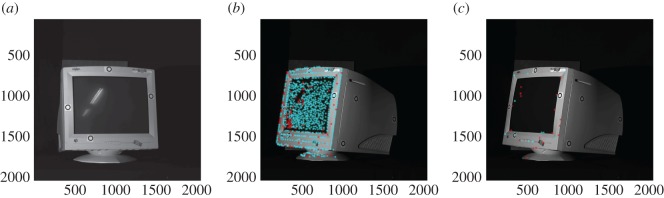
First view (*a*) and second view (*b*,*c*) of CRT monitor. Stars indicate keypoints detected in the second view point cloud by ISS (*b*) and Harris 6D (*c*) detectors. The keypoints repeated between the first and second views are shown in cyan, and those not repeated in the first view are shown in red.

In each view, there were on average 2540 ISS keypoints, whereas there were 315 Harris 6D keypoints per view on average. Harris keypoints tend to be associated with corners of the object, whereas ISS keypoints tend to detect points with significant curvature relative to the neighbourhood, capturing both points and edges in the object. The average repeatability of the ISS keypoint descriptor from one view to another was 90%, whereas that of the Harris 6D keypoint detector was 28%. This is illustrated in figure 15*b* for the ISS detector and in figure 15*c* for the Harris 6D detector. The first view is shown in figure 15*a* as a reference to confirm that there is a significant overlap between the two views in the illustration. It should be pointed out, however, that the criterion for a successful match of keypoints across views is that the Euclidean distance lies below a threshold value of 10 mm. The repeatability value for ISS may therefore be misleadingly high, because the high keypoint density means that a significant number of unmatched keypoints can appear to be matched when using this criterion alone. Nevertheless, in view of the low repeatability value for the Harris 6D detector, the ISS keypoint detector was used for further analysis in the following section.

### 7.2. Repeatability of descriptors across views

**Figure 16.**
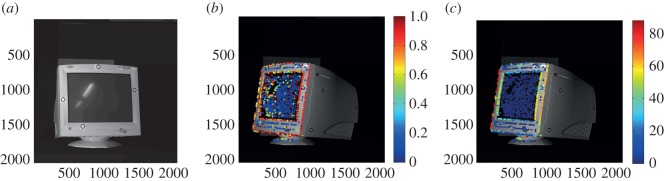
The Euclidean distance between the best keypoint descriptor match for close keypoints between two views. (*a*) first reference view, (*b*) SHOT descriptor match distance in the second view and (*c*) FPFH descriptor match distance in the second view, calculated at keypoints extracted using the ISS detector.

At the keypoints identified in the previous subsection, signature of histograms of orientation (SHOT) [29] and fast point feature histogram (FPFH) [30] keypoint descriptors were calculated in a neighbourhood of *R*_keypoint_=10 mm radius sphere. The SHOT descriptors were calculated using pcl::SHOTEstimation::compute function, whereas FPFH descriptors were calculated using pcl::FPFHEstimation::compute function. For each keypoint in a second view, the keypoint with the closest descriptor (in Euclidean distance) was found from the first view (shown in figure 16*a*) within a neighbourhood of 10 mm radius. Thus, the best matched keypoint close in 3D distance was found for each keypoint and the distance obtained for each keypoint is shown in figure 16*b* for SHOT descriptor and figure 16*c* for FPFH descriptor. As can be seen, the best matched keypoints (blue) are in the smoothly varying mostly planar regions, whereas the worst matched keypoints (red) are where the curvature is high along the edges of the object. This is a fundamental failure in these type of objects where high-curvature points cannot be matched from one view to another.

**Figure 17.**
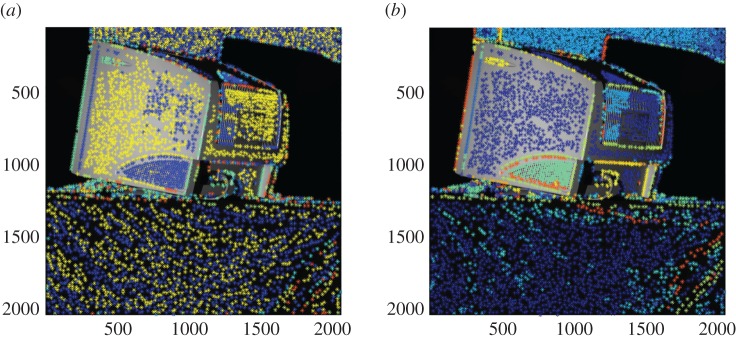
Grouped (*a*) SHOT descriptor (*b*) FPFH descriptor calculated at keypoints extracted using the ISS detector in a scene containing the CRT monitor with little background clutter.

In a scene containing the CRT monitor with little background clutter (figure 8), both SHOT and FPFH keypoint descriptors were calculated and grouped into 10 groups by the MATLAB kmeans algorithm[31]. The keypoints identified from the scene colour coded into groups are shown in figure 17*a* for SHOT and figure 17*b* for FPFH descriptors. As can be seen, the keypoints that have the same visual and 3D characteristics belong to the same group (i.e. all keypoints on an edge/all keypoints on a smooth surface). However, the inability of descriptors at crucial points/edges to repeat with view changes poses a serious problem in recognizing these types of objects.

### 7.3. Results of object recognition

Despite poor repeatability across views described above, the keypoint descriptors computed at ISS keypoints of the scene containing the CRT monitor with little background clutter (figure 8) were matched to the keypoint descriptors of the model (aggregated keypoints and descriptors from seven views of the isolated CRT monitor). A match was defined by the Euclidean distance between descriptors normalized using Frobenius norm being smaller than 0.25. This produced over 6 million correspondences for both SHOT and FPFH descriptors.

Subsequently, the matches were passed onto a Hough transform-based clustering algorithm in PCL to determine the pose using function pcl::Hough3DGrouping::recognize. A Hough bin size of 40 mm with a threshold of 10 votes was used. The algorithm identified over 100 potential poses; however, none of these matched the actual pose as determined by the maximum-likelihood algorithm.

To check the complete PCL object recognition pipeline, a pose that was used to generate the model was also used as a scene. As expected, the program then made perfect keypoint descriptor matches (i.e. a large subset of the same keypoints with the same descriptor appear in both the scene and the model) and in turn easily recognized the object pose as that with the most votes in the Hough space. Thus, it could be concluded that the problem lies in computing descriptors at keypoints that are sufficiently unique in the object. For the types of objects used in this study, and the two chosen keypoint detectors, there do not appear to exist keypoints that can be extracted stably across views. Furthermore, the resulting keypoint descriptors are not unique enough to be used in matching.

## 8. Conclusion

A maximum-likelihood-based object recognition and localization algorithm has been presented. Compared to ICP approaches, the algorithm is computationally efficient, and compared to Hough transform approaches, it has no significant memory requirements. The algorithm involves constructing a PDF by considering the matching of surface patches of the model to the surface patches of the scene. The surface patches are identified by segmenting the image and identifying smoothly varying surface segments. A gold standard model was created using several views of a marked model and combining the segmented surfaces to create a complete 3D part.

Once the PDF is created as the product of the matching probabilities over all patches in the model, object localization is achieved by identifying the position of the maximum of the log-likelihood function with a standard gradient-based optimization algorithm. The global optimization is conducted by broadening the spread of the PDF as well as decreasing the baseline probability to increase the number of matching surface patches. The value of the likelihood function at its peak can be used to decide on the presence or absence of the part in the scene, so that object recognition is a built-in feature of the proposed method. After approximate estimation of the pose with a wide spread of the PDF, a fine localization is conducted by narrowing the spread. The complete algorithm thus provides a unified approach to object recognition and localization by removing the need for a separate time-consuming ICP algorithm to refine the object pose. Evidence of the performance of the algorithm was provided for an illustrative part recognition problem, both in isolation and within a cluttered scene. Datasets from two further samples with widely different geometries were also analysed by the proposed algorithm; in both cases, this was able to determine successfully both the initial approximate estimate of the pose and the refined pose estimate.

We have provided evidence that the same algorithm can be used to recognize objects within a scene. The method has been successful for the limited number of objects used in this experiment, indicating the potential use of the algorithm for object recognition.

The performance of several previously published 3D object recognition and pose-estimation algorithms, based on keypoint descriptors and Hough transforms, has also been investigated using the same datasets as for the maximum-likelihood method. However, none of the algorithms tested were able to recognize the pose of the object due to the presence of a large number of non-unique descriptor matches in the object of interest. Therefore, we conclude that for objects with relatively few unique keypoints, but with large surface areas, the maximum-likelihood algorithm presented here is better suited to the problem of object recognition and pose estimation than these alternative approaches.

## Data accessibility

Data available from the Dryad Digital Repository: http://dx.doi.org/10.5061/dryad.37k3n [32].
